# An intervention targeting fundamental values among caregivers at residential facilities: effects of a cluster-randomized controlled trial on residents’ self-reported empowerment, person-centered climate and life satisfaction

**DOI:** 10.1186/s12877-016-0306-2

**Published:** 2016-07-07

**Authors:** Charlotte Roos, Marit Silén, Bernice Skytt, Maria Engström

**Affiliations:** Department of Health and Caring Sciences, Faculty of Health and Occupational Studies, University of Gävle, Kungsbäcksvägen 47, 801 76 Gävle, Sweden; Department of Public Health and Caring Sciences, Uppsala University, Uppsala, Sweden

**Keywords:** Empowerment, Experimental design, Living in dignity, Person-centered climate, Residential facilities for older people, A sense of well-being, Life satisfaction

## Abstract

**Background:**

In Sweden the national fundamental values for care of older people state that care should ensure that they can live in dignity and with a sense of well-being. Our hypothesis was that a caregiver intervention targeting the national fundamental values would improve perceived empowerment, person-centered climate and life satisfaction among older people living in residential facilities.

**Methods:**

The study was a cluster-randomized controlled trial with a pre- and one post-test design, conducted in 27 units (17 study units) at 12 residential facilities for older people in five municipalities in central Sweden. The units in each municipality were randomly assigned to intervention or control group. The caregiver intervention was carried out using an interpretative approach with eight guided face-to-face seminars, where self-reflection and dialogue were used. Data were collected using questionnaires. The number of residents was 43 (78 %) in the intervention group and 37 (71 %) in the control group. The Chi-square test and Mann-Whitney U-tests were performed to detect differences between groups and Wilcoxon signed rank tests to explore differences in change over time within groups. Furthermore, generalized estimating equation (GEE) models were used to study effects of the intervention controlling for clustering effects.

**Results:**

Primary outcome measures were empowerment, person-centered climate and life satisfaction. In the intervention group, improvements at follow-up were found in residents’ self-reported empowerment (*n* = 42; *p* = 0.001, Median difference 4.0, 95 % CI 1.5;6.0), person-centered climate (*n* = 42; *p* ≤0.001, Median difference 8.0, 95 % CI 4.5;11.4) and life satisfaction regarding the factor quality of everyday activities (*n* = 40; *p* = 0.033, Median difference 9.7, 95 % CI 1.0;21.9) while disempowerment decreased (*n* = 43; *p* = 0.018, Median difference -1.3, 95 % CI -2.0;0.0). In the control group person-centered climate decreased (*n* = 37; *p* = 0.002, Median difference -8.5, 95 % CI -13.6;-3.0) and quality of everyday activities (*n* = 36; *p* = 0.012, Median difference -11.6, 95 % CI-21.7;-3.4). Change over time between groups was significant for empowerment (*p* = 0.001, Median difference 6.0, 95 % CI 3.0;9.0), disempowerment (*p* = 0.006, Median difference -2.0, 95 % CI -4.0;-1.0) and person-centered climate (*p* ≤ 0.001, Median difference 16.0, 95 % CI 9.7;23.0) and for life satisfaction regarding the factor quality of everyday activities (*p* = 0.002, Median difference 22.1, 95 % CI 8.2;37.4). Results of GEE confirmed earlier results; revealed interaction effects for empowerment (parameter estimate -5.0, 95 % CI -8.3;-1.8), person-centered climate (parameter estimate -16.7, 95 % CI -22.4;-10.9) and life satisfaction regarding the factor quality of everyday activities (parameter estimate -25.9, 95 % CI -40.3;-11.5).

**Conclusion:**

When the Swedish national fundamental values were put into practice increases in empowerment, person-centered climate and quality of everyday activities were found among older people with intact cognitive ability living in residential facilities. Limitations to consider are the differences between the two groups at baseline, drop-outs and that neither the data collector nor the outcome assessors were blinded to group assignment of participants.

**Trial registration:**

The study was registered in ISRCTN92658034 in January 2013.

## Background

The purpose of fundamental values in care is to stress and clarify important values that are worth striving for [1, 2]. Values can be defined as basic convictions concerning what is right, good and desirable [3]. Moreover, values guide our actions, as they can provide support for caregivers in performing and developing care. Yet another reason for clarifying values in care is to minimize the risk that they will be disregarded and to protect persons who are humiliated or badly treated [1]. Living in dignity and with a sense of well-being have been central values in care of older people in Sweden since 2011, when the national fundamental values were legislated in The Social Services Act, which regulates care of older people. The national fundamental values state that care of older people should ensure that they live in dignity and with a sense of well-being. Living in dignity requires respect for the values; personal integrity, self-determination, participation, individualized care of good quality and kind treatment. A sense of well-being requires respect for the values; meaningfulness and safety [4]. The Swedish National fundamental values are related to a number of concepts, for example, empowerment, person-centered climate and life satisfaction.

Empowerment is described as a process of promoting and enhancing people’s ability to meet their own needs and to mobilize the resources necessary to feel control over their lives. Outcomes of empowerment are self-efficacy, a sense of mastery, a sense of control and improved quality of life [5]. Empowering care, which helps people maintain control over their lives promotes well-being [6] and is also an important predictor of quality of life [7]. Older people living in residential facilities emphasize the importance of maintaining their physical abilities [8, 9] for experiencing a sense of control, which has a great influence on having a sense of dignity [10] and well-being [11] in life. Absence of empowerment is described as powerlessness, helplessness, hopelessness and loss of a sense of control over one’s life [5]. Dis-empowerment is defined as an active process of preventing people from maintaining control over their lives, which leads to increased dependence [12]. In previous studies, older people have described the environment in residential facilities as disempowering because it does not strengthen their self-determination, participation or control in daily life [13].

A person-centered climate includes feeling welcome, safe and familiar and being able to create and maintain social relations [14]. Concerning maintaining social relations, residents describe that relations with family and friends are important for experiencing dignity [9, 15–17] and well-being [18] in life. However, due to their advanced age, residents experience having few family members and friends [9, 15, 19] and further that residential facilities have few options for social relationships with other residents [15, 19] and staff, which leads to a feeling of being abandoned [8, 17]. A person-centered climate has to support the personhood of people living in a residential facility, as this is important for a feeling of familiarity [14]. Residents describe experiencing a strong sense of dignity in life when there is agreement between their own values and staff actions that convey these values but such experiences are often lacking in residential facilities [15].

The climate of a residential facility further includes an interplay between the physical environment, people acting and being in the environment and the organizational philosophy of care [20]. In dementia care settings, the presence or absence of staff is highly influential in creating a person-centered climate, which in turn influences residents’ well-being. When staff members share both place and moments with residents, they are observed to make efforts to see and acknowledge residents, welcome them and to make an effort to participate in residents’ everyday life. When staff members share both space and moments with residents, the climate is further characterized by a feeling of at-homeness, where residents show signs of well-being [21]. The importance of a person-centered climate and environment in promoting a sense of well-being among residents has also been highlighted in other studies [22, 23]. Despite these positive outcomes, staff members sometimes focus on routines and tasks rather than the person [24], and focusing on routines has a negative impact on nursing practice, which loses its humanity and violates the person’s integrity [25].

Not only do older people living in residential facilities experience the positive outcomes of living in a context of an empowering and person-centered climate, studies have also described negative outcomes for residents when care is experienced as disempowering and when the climate is not person-centered. Nonetheless, few intervention studies in residential facilities have attempted to test the outcomes of putting empowerment and a person-centered approach into practice. Studies trying to put working methods to enhance empowerment into practice have mostly investigated staff perceptions and experiences [26–29]. These studies found that empowerment interventions do help in putting a person-centered approach into practice. Studies attempting to put person-centered care into practice have primarily been performed in dementia care settings and have mostly examined staff members’ perceptions. One study found that when implementing national guidelines for person-centered care in a dementia care setting, there were no significant differences in staff ratings concerning the overall person-centered climate at follow-up, but there was a significant increase in perceived hospitality [26]. In another study aimed at investigating the effects on care provision and caring climate among nursing assistants before and after an intervention targeting a palliative care approach at residential facilities, no effects were observed for person-centered care or the caring climate [30].

There is a lack of intervention studies in which a person-centered approach is put into practice in residential facilities for older people with intact cognitive ability. Furthermore there is a lack of studies, in these settings, investigating residents’ perceptions of empowerment, person-centered climate and life satisfaction. Based on the limited amount of previous research, the aim of the present study was to examine residents’ perceptions of empowerment, person-centered climate and life satisfaction before and after a caregiver intervention concerning the Swedish national fundamental values. A further aim was to investigate whether there were any differences in change over time in these variables between an intervention group that received the intervention and a control group. Our hypothesis was that a caregiver intervention concerning the Swedish national fundamental values would improve perceived empowerment, person-centered climate and life satisfaction among residents.

## Methods

### Study design

The study was a cluster-randomized controlled trial with a pre- and one post-test design.

### Units, participants, setting and procedures

#### Units

**Fig. 1:**
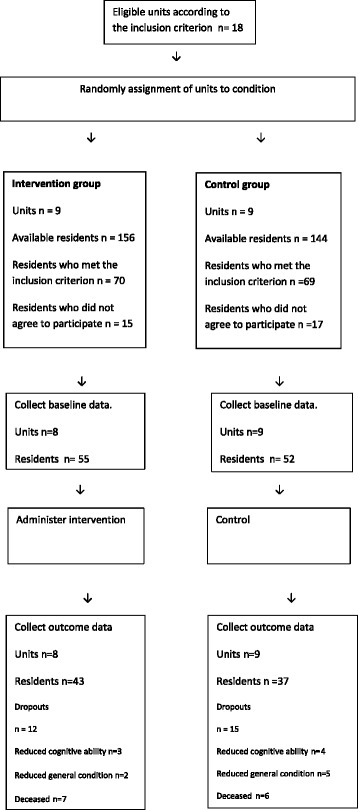
Flow chart of progress of units and participants

The study was conducted in five municipalities in central Sweden. The municipalities were recruited using a convenience sample. The executive managers in eight municipalities were approached; five agreed and gave their written consent to participate in the study. Written information about the study was then sent to managers at residential facilities in the municipalities, according to the inclusion criterion. The inclusion criterion for residential facilities was that residents should live permanently in the facility, and the exclusion criterion was that the facilities should not be specialized in dementia care. Managers at 12 residential facilities (27 units), varying in terms of location (urban: 58 %; rural: 42 %), volunteered and gave their oral consent to participate in the study. All facilities were publicly funded. Before randomization, the first author together with the manager at the residential facility identified units that shared staff. These were treated as one study unit and thereby units that shared staff could not end up in different groups. For other units at the same residential facility, not sharing staff, they could end up in either group. The study units in each municipality were then randomized to the intervention group (9 study units) or the control group (9 study units). A table of random numbers was used for randomization [31]. Randomization took place after the residential facilities had been recruited, based on the inclusion and exclusion criteria, but before the baseline data were collected (Fig. 1).

#### Participants

The number of residents living in the units was 156 in the intervention group and 144 in the control group. Inclusion criteria for residents were being able to complete a questionnaire by oneself or with assistance from the first author or a research assistant, hereafter labeled assessor, and having resided for more than 2 weeks at the facility. Exclusion criteria for residents were diagnosed dementia and/or reduced cognitive ability, with difficulties understanding the questions. The registered nurses at the residential facilities reported to the first author the number of residents with diagnosed dementia and the number of residents with, as they estimated, reduced cognitive ability. The first author or the assessor asked all residents who met the inclusion criteria if they would like to participate. In the intervention group 55 of the 70 (79 %) residents who met the inclusion criteria agreed to participate, and in the control group, 52 of the 69 (75 %) residents who met the inclusion criteria agreed to participate. During the study 12 of the 55 (22 %) residents in the intervention group and 15 of the 52 (29 %) residents in the control group dropped out. The most frequent reason for dropping out was that the resident was deceased. The number of residents who completed the questionnaire at baseline and follow-up was 43 (78 %) in the intervention group and 37 (71 %) in the control group; see Fig. 1.

Written information regarding informed consent from the residents, information about the study, the questionnaire and the response alternatives were written in a large font to facilitate reading. Nevertheless all residents needed assistance with reading the questionnaire, and therefore received help from the first author or the assessor. The questions and the response alternatives were then read out loud for the residents. The response alternatives were written on a separate card in a large font to further facilitate reading. All of the residents chose to perform the data collection in their apartments at the facility. Prior to the questionnaire, the residents received information from the first author or the assessor. The information explained the aim of the study, that participation was voluntary, that they could withdraw from the study at any time and that the data would be treated confidentially. The residents also signed an informed consent form.

#### Setting

The number of residents in each unit ranged from 7–16, which is representative of the unit sizes in Sweden. When averaged for the 27 units there were 10.4 residents/unit in the intervention group and 12.0 residents/unit in the control group. The facility provided a one-room apartment with a kitchenette and a private bath and toilet. The furniture belonged to the residents themselves. The dining area was communal. The municipality was responsible for all facilities and the care and service provided there. The facilities were staffed by nurse assistants (NA) and registered nurses (RN) around the clock. The facilities used the working methods “contact person” and “individualized care plans.” NAs, in their role as “contact person”, have a particular responsibility for planning, providing and evaluating care for each resident. As “contact person”, NAs are also responsible for making an “individualized care plan” for each resident. The plan describes how the residents want their daily care to be carried out.

### Data collection

Data were collected using a questionnaire at baseline and follow-up (2 months after intervention). As the intervention was divided in two periods (Period I: 2013-01-01 – 2013 -06-30; Period II: 2013-09-01–2014-02-28) baseline data were collected in December 2012 and in August 2013. Follow-up data were collected in August 2013 and in April 2014. Half of the units from the intervention and control groups participated in period I and the other half in period II. The first author and the assessor organized the data collection and distributed the questionnaires personally; the data collectors were not blinded to which group the residents belonged to. The assessor was selected for having considerable experience of working as an NA in residential facilities for older people, good verbal skills and a sociable personality. The assessor was trained by the first author prior to data collection. There were no dependencies between the first author, the assessor and the residents prior to the study. At data collection, both the first author and the assessor went to the same residential facility. The first author was therefore available for the assessor during data collection.

### Measures

#### Characteristics of participants

The personal characteristics data collected were age, gender and period of residence. Furthermore, the EQ-5D questionnaire [32] consisting of five items was used to measure mobility, self-care, usual activities, pain/discomfort and anxiety/depression. The questions are multiple-choice questions and the response alternatives range from 1 (no problems) to 3 (severe problems). The questionnaire further includes the “EuroQol thermometer,” a scale ranging from 0 (worst imaginable health) to 100 (best imaginable health) on which the participants rate their health status [32].

#### Empowerment

Empowerment was measured using the Patient Empowerment Scale [12] consisting of 40 items measuring two factors: empowerment and disempowerment. The response alternatives are multiple-choice and range from 0 (never) to 2 (often). The total score for empowerment can range from 0 to 40 and the total score for disempowerment can range from 0 to -40. Satisfactory psychometric properties have been reported: Cronbach’s alpha values between 0.74 -0.87 for empowerment and between 0.65-0.87 for disempowerment. See the result section for the values in the present study. The Patient Empowerment scale was translated into Swedish and the procedure for translating the scale involved the three established steps suggested by Polit and Beck [31]. First, the research group translated the English version into Swedish. Second, a professional translator carried out the back translation of the Swedish version into English. Third, the research group conducted a thorough comparison of the three versions of the scale. As the Patient Empowerment Scale had not been assessed in a population of residents living in residential facilities for older people, the relevance of the questionnaire was assessed in that context prior to conducting the study. The questionnaire was completed by five residents in a municipality that did not take part in the study. In addition to answering the questionnaire, the residents also commented on the feasibility of the questionnaire. No subsequent changes were made in the questionnaire.

#### Person-centered climate

Person-centered climate was measured using the Swedish language Person-centered Climate Questionnaire - patient version (PCQ-P) [33], consisting of 17 items measuring three subscales: a climate of safety, a climate of everydayness, and a climate of hospitality. Response alternatives are presented on a 6-point Likert-type scale ranging from 1 (No, I disagree completely) to 6 (Yes, I agree completely). Sum scores are used for the subscales and for the total score of the scale. The total scale can range from 17 (minimally person-centered climate) to 102 (maximally person-centered climate). Satisfactory psychometric properties have been reported; Cronbach’s alpha values 0.93 for the total scale and for the subscales: safety 0.94, everydayness 0.82 and hospitality 0.64. See the result section for values in the present study.

#### Life satisfaction

Life satisfaction was measured using the Life Satisfaction Questionnaire (LSQ), constructed to measure life satisfaction [34]. The questionnaire consists of 34 items measuring six factors: quality of close friend relations, physical symptoms, quality of everyday activities, quality of family relations, socio-economic situation and sickness impact. Item 34 – How do you perceive your overall quality of life? – is used as a single-item measure. Response alternatives for the LSQ range from 1 (overall negative) to 7 (overall positive), with high scores indicating high satisfaction. Satisfactory psychometric properties have been reported; Cronbach’s alpha values for the factors: quality of close friend relations 0.90, physical symptoms 0.75, quality of everyday activities 0.92, quality of family relations 0.87, socio-economic situation 0.62 and sickness impact 0.85. See the result section for values in the present study.

### Intervention

The Swedish national fundamental values were central to the caregiver intervention, which aimed at a) providing a shared understanding among caregivers of the national fundamental values and further to provide a shared understanding of the working methods “contact person” and “individualized care plans” in relation to the national fundamental values, b) identifying, on the individual, group and organizational level, difficulties, possibilities, weaknesses and strengths when the national fundamental values and working methods “contact person” and “individualized care plans” are put into practice, c) exploring and trying to understand residents’ perceptions and experiences of the national fundamental values and d) developing and putting into practice an improvement plan intended to increase residents’ sense of dignity and well-being in life.

In the intervention a total of 124 caregivers from the intervention units participated in eight three-hour face-to-face seminars, with an average attendance of 6.7 seminars, ranging from 3 to 8. The caregiver intervention was carried out over a 6-month period by the first author, who guided the seminars. All seminars followed a protocol. The seminars took place every 2 weeks. Furthermore, the first author visited the units every 2 weeks, for approximately three hours, to answer questions about the intervention. After eight seminars (4 months), the first author continued to visit the units every 2 weeks for two additional months to offer support to the staff as they put the improvement plan into practice. The first author never visited the control group. The Swedish National Fundamental Values were not discussed, in any formal way, in the control group.

The content of the seminars was as following: *Dignity* (self-determination, respect for personal integrity, participation, individualized care of good quality and kind treatment), *well-being* (meaningfulness and safety), *working methods* (contact person and individualized care plans) (Seminar 1–5), and *development* and *evaluation* of an *improvement plan* (Seminar 6–8); see Table 1. Between seminars 1–5, the caregivers had an individual assignment connected to the residents for whom they were the contact person, the aim of which was to explore and try to understand the residents’ perspective. The assignment was constructed in three steps: a) to reflect on what the caregivers themselves thought was important for the residents concerning the values discussed at the seminar, b) to have a dialogue with the residents concerning what was important in daily life concerning these values, and c) to reflect on what the caregivers could do to enable realization of the values in residents’ daily life. The assignments were reflected on at the following seminar and were later used when developing the improvement plan for the unit.

**Table 1:** Aim, content of each seminar and working methods in the intervention

Aim	Seminar/content	Working methods
To provide a shared understanding among staff of the national fundamental values; living in dignity and with a sense of well-being and associated values. To identify difficulties, possibilities, weaknesses and strengths concerning the national fundamental values on the individual, group and organizational level. To explore and try to understand residents’ perceptions and experiences of the national fundamental values and associated values.	1. Self-determination 2. Respect for personal integrity and participation 3. Safety and meaningfulness 4. Individualized care and kind treatment	At seminars: Self-reflection and dialogue Between seminars: Assignment connected to resident
To provide a shared understanding among staff of working methods “contact person” and “individualized care plan” and their content in relation to national fundamental values. To identify difficulties, possibilities, weaknesses and strengths concerning working methods “contact person” and “individualized care plans”, in relation to national fundamental values, on the individual, group and organizational level.	5. Contact person and individualized care plan	At seminar: Self-reflection and dialogue Between seminars: Read an individualized care plan and identify if and how the national fundamental values are expressed in the plan. Together with a resident, update the care plan in relation to the national fundamental values.
To provide a shared understanding among staff of improvements needed to increase residents’ sense of dignity and well-being in relation to identified difficulties, possibilities, weaknesses and strengths. To develop and implement an improvement plan.	6. Identify improvements and develop an improvement plan	At seminar: Self-reflection and dialogue Between seminars: Implement the improvement plan
To provide a shared understanding among staff of what they perceive as good quality of care in relation to the national fundamental values. To evaluate and further develop the improvement plan.	7. Good quality of care and evaluation and further development of the improvement plan	At seminar: Self-reflection and dialogue Between seminars: Implement the improvement plan
To provide a shared understanding among staff and the manager of how to proceed with the improvement plan. To make staff aware of the results from the baseline measures.	8. Evaluation of improvement plan and feed-back from baseline measures	At seminar: Dialogue with manager about how to proceed with the improvement plan. Dialogue about the baseline measures.

The Medical Research Council [35] has described the main stages of a complex intervention as development, feasibility/piloting, evaluation and implementation. In each stage different key functions and activities are outlined. The focus for the present intervention was on the evaluation stage where the effectiveness of the intervention was assessed and steps were taken to understand the change process.

The theoretical basis for the intervention was an interpretative approach, as described by Sandberg and Targama [36, 37]. As the intervention followed an interpretative approach self-reflection and dialogue were used as working methods at the seminars. According to Sandberg and Targama [36, 37] the way in which work is understood constitutes the core of staff members’ actions in the organization, as actions are not primarily guided by factors such as rules and instructions, but by how the situation, rules and instructions are understood. The way in which staff members understand the work situation is the way in which they will act. Actions can be understood in different ways, and the more complex actions are the more possibilities there are to understand them differently. Accordingly, it is important to develop and maintain a shared understanding among staff of the actions in the organization. By changing one’s understanding of one’s actions, one can learn to change, and based on this new competence can be developed and maintained. The most central prerequisite for achieving learning through change of understanding is through reflection. By reflecting on our understanding of actions, it is possible to become aware of our understanding. Reflection can be performed through self-reflection [36]. In the intervention, the caregivers used self-reflection when reflecting on questions such as: “How do I understand the value of self-determination?” Self-reflection was then shared in the group, the aim being to increase awareness of the different understandings in the group. Reflection can also be performed through dialogue with others. Dialogue develops the collective competence of the group that is cooperating. In order to cooperate, it is important to develop a shared way of understanding work [36, 37]. In the intervention, the caregivers used dialogue to deal with questions such as: “How do we understand the value of self-determination in our group and what is our shared understanding of it?” To further identify staff members’ understanding of work, their experiences of their actions were worked through in a reflective process. Questions such as “What actions do you perform in daily care to promote residents’ self-determination?” were used in self-reflection. Self-reflection was then shared in the group, and the caregivers further had a dialogue concerning their shared understanding of what actions in daily care promote residents’ self-determination. This approach as described by Sandberg and Targama [36, 37] is also in line with other theories of learning (see for example Säljö [38] and Ramsden [39]).

After creating a shared understanding of the national fundamental values having a sense of dignity and well-being in life, caregivers reflected on the difficulties, possibilities, weaknesses and strengths associated with these values in relation to their current actions on an individual, group and organizational level in order to identify improvements. Identified difficulties and weaknesses were used as a premise for improvements in the improvement plan. Identified possibilities and strengths were used to facilitate the improvements.

### Data analysis

Due to the small sample and fairly low intracluster coefficients for most variables, non-parametric statistics were first used for analysis on resident level. To explore differences in change over time within groups, Wilcoxon signed rank tests were used. To explore differences in baseline data and in change over time between groups, Mann-Whitney U-tests were used. For confidence interval (CI) we used related-samples and independent-samples Hodges-Lehman median (Md) difference. However, even if the intracluster coefficients for most variables were fairly low we also tested the results using linear generalized estimating equation (GEE) models to control for potential correlations among residents within the same units, i.e. clustering effects to see whether theses analysis changed our results or not. In the GEE models, the independent variables were main effects for group (intervention and control groups) and time (baseline and follow-up) as well as interaction effect (time*group). Intraclass correlation coefficient (ICC) for clustering effects was calculated using Linear Mixed Models. For all variables, when ≤35 % of the items were not completed in a factor, these missing values were replaced with the individual’s mean for the factor. When more items were missing the factor was excluded. All residents were analysed in the groups to which they were randomized if they had complete/available data (baseline – follow-up). Scale distribution and normality were explored using measures of skewness and visual examination of histograms and Q-Q plots. Reliability measured as internal consistency was explored using Cronbach’s alpha. All statistical analyses were performed using SPSS statistics 22. *P*–values <0.05 (two-tailed) were regarded as statistically significant.

## Results

A total of 80 residents answered the questionnaire at baseline and follow-up (intervention group 43 and control group 37), resulting in a response rate of 78 % in the intervention group and 71 % in the control group; see Fig. 1. Most participants were female: intervention group 27 (63 %) and control group 24 (65 %). The mean age was 86.8 (standard deviation [SD] 7.3) and 87.1 (SD 8.2) in the intervention and control groups, respectively. The mean of period of residence was 27.1 months in the intervention group (SD 36.7) and 31.75 months in the control group (SD 29.4). There were no significant differences in the personal characteristic parameters between groups (see Table 2). Regarding outcome measures, there were significant differences between the groups for the factors quality of daily activities (*p* = 0.046, Md difference -10.2, 95 % CI -25.2; 0.0), sickness impact (*p* = 0.028, Md difference -9.5, 95 % CI -16.7;0.0) and hospitality (*p* = 0.034, Md difference -2.0, 95 % CI -3.0;0.0). The control group rated these factors higher than the intervention group did at baseline. Between participants and drop-outs, there were significant differences in personal characteristic parameters regarding anxiety and depression (*p* = 0.047), in that the drop-outs rated these factors lower than the participants did. Furthermore, there were differences in empowerment (*p* = 0.017, Md difference 6.0, 95 % CI 1.0;10.0) and disempowerment (*p* = 0.020, Md difference 2.0, 95 % CI 0.0;4.0) in that drop-outs rated both factors higher than the participants did.

**Table 2:** Characteristics of the sample in intervention and control group at baseline

	Intervention group *n* = 43	Control group *n* = 37	*p*-value
Gender			0.847
Male/female	16/27	13/24	
Age/year			0.721
Median (Q1-Q3)	89.0 (82–92)	88.0 (83–93)	
Min-max	65–99	68–104	
Period of residence/Months			0.147
Median (Q1-Q3)	13.0 (5–36)	23.0 (12–46)	
Min-max	1–156	1–120	
EQ-5D			
Mobility			0.537
No problems in walking about	1	2	
Some problems in walking about	26	24	
Confined to bed	16	11	
Self-care			0.175
No problems with self-care	7	5	
Some problems washing or dressing	31	25	
Unable to wash or dress	5	7	
Usual activities			0.661
No problems with performing usual activities	22	21	
Some problems with performing usual activities	14	13	
Unable to perform usual activities	6	3	
Pain/discomfort			0.237
No pain or discomfort	16	20	
Moderate pain or discomfort	15	12	
Extreme pain or discomfort	12	5	
Anxiety/depression			0.351
Not anxious or depressed	19	16	
Moderately anxious or depressed	19	16	
Extremely anxious or depressed	5	5	
Health state			0.118
Median (Q1-Q3)	50 (50–80)	72.5 (50–88.8)	
Min-max	25–100	0–100	

*SD* Standard deviation, *Q* Quartile, For age, period of residence and EQ-5D Mann-Whitney U-tests have been used and for gender Chi^2^ test has been used. For usual activities there is 1 missing data in the intervention group and for health state there is 1 missing data in the control group

### Effects on empowerment

The intervention group had higher scores on empowerment (*p* = 0.001) and lower scores on disempowerment (*p* = 0.018) at follow-up compared to baseline. In the control group, no significant differences at follow-up compared to baseline were found for empowerment and disempowerment. Change over time between groups was significant for empowerment (*p* = 0.001) and disempowerment (*p* = 0.006); see Table 3 for Md differences and 95 % CIs.

**Table 3:** Baseline and follow-up scores of empowerment, person-centered climate (PCQ-P) and life satisfaction (LSQ)

Variables	Intervention group		Differences between baseline and follow-up	Within group		Control group		Differences between baseline and follow-up	With in group		Differences in change over time between groups	ICC for clustering effect using LMM
	Baseline	Follow-up	Md (95 % CIs)^a^	*p*-value^b^	α	Baseline	Follow-up	Md (95 % CIs)^a^	*p*-value^b^	α	Md, (95 % CI)^c^, *p*-value^d^	
Empowerment			4.0 (1.5;6.0)	**0.001**	0.72			-2.0 (-5.0;0.5)	0.091	0.85	6.0 (3.0;9.0) **0.001**	0,0768
• Md (Q1-Q3)	26.0 (21–29.3)	30.0 (24–33)				23.0 (20–31.8)	20.5 (16–28.9)					
• Min-max	16–35	8–40				10–38	11–36					
Disempowerment			-1.3 (–2.0;0.0)	**0.018**	0.65			1.0 (–0.5;2.5)	0.115	0.85	-2.0 (-4.0;-1.0) **0.006**	0,0425
• Md (Q1-Q3)	3.0 (1–6)	1.0 (0–4)				2.0 (0–7)	4.0 (1–7)					
• Min-max	0–9	0–18				0–13	0–16					
Total PCQ-P			8.0 (4.5;11.4)	**≤0.001**	0.86			-8.5 (-13.6;-3.0)	**0.002**	0.90	16.0 (9.7;23.0) **≤0.001**	0,1164
• Md (Q1-Q3)	83.0 (75–93)	94.0 (84.9–100)				90.0 (78.7–96)	77.0 (68.5–90)					
• Min-max	46–102	47–102				46–102	35–102					
Safety			3.5 (1.5;5.5)	**0.001**	0.84			-3.4 (-6.0;-0.8)	**0.013**	0.87	7.0 (3.8;10.0) **≤0.001**	0,0568
• Md (Q1-Q3)	50.0 (47–57)	56.0 (52–58)				55.0 (49–59)	50.0 (46.5–57.8)					
• Min-max	29–60	34–60				36–60	23–60					
Everydayness			1.5 (0.0;3.0)	**0.009**	0.81			-2.0 (-4.0;0.0)	**0.028**	0.76	4.0 (1.3;6.0) **0.001**	0,0917
• Md (Q1-Q3)	20.0 (16–24)	23.0 (19–24)				21.0 (16–24)	17.0 (13–21.5)					
• Min-max	7–24	5–24				4–24	4–24					
Hospitality			3.0 (1.5;4.5)	**≤0.001**	0.40			-2.8 (-4.0;-1.5)	**≤0.001**	0.49	6.0 (4.0;8.0) **≤0.001**	0,1526
• Md (Q1-Q3)	13.0 (8–16)	16.0 (14–18)				15.0 (12–18)	11.0 (8–14.5)					
• Min-max	8–18	8–18				6–18	6–18					
Quality of everyday activities			9.7 (1.0;21.9)	**0.033**	0.88			-11.6 (-21.7;-3.4)	**0.012**	0.93	22.1 (8.2;37.4) **0.002**	0,2084
• Md (Q1-Q3)	36,7 (16.7–59.7)	59.5 (26.2–77.6)				46.6 (23.4–80.3)	31.0 (20.2–60.2)					
• Min-max	14.3–89.8	14.3–98.0				14.3–100	14.3–98.0					
Sickness impact			1.8 (-3.6;6.0)	0.516	0.66			-2.4 (-7.1;2.4)	0.329	0.76	4.8 (-2.4;9.5) 0.235	0,0485
• Md (Q1-Q3)	69.0 (57.1–83.3)	71.4 (59.5–85.7)				81.0 (67.9–92.9)	81.0 (67.9–88.5)					
• Min-max	33.3–100	16.7–97.6				40.5–100	38.1–100					
Physical symptoms			0.0 (-3.1;3.1)	0.911	0.43			-1.0 (-3.1;1.0)	0.333	0.64	2.0 (-2.0;6.1) 0.512	0,0285
• Md (Q1-Q3)	87.8 (81.6-93.9)	91.8 (81.6-98)				91.8 (83.7-99)	91.8 (84.7-98)					
• Min-max	63.3–100	59.2–100				71.4–100	59.2–100					
Socio-economic situation			0.0 (-5.4;4.8)	0.962	0.79			-4.2 (-8.9;0.0)	**0.047**	0.79	3.6 (-3.6;10.7) 0.217	0,0134
• Md (Q1-Q3)	71.4 (57.1–82.1)	71.4 (64.3–78.6)				71.4 (64.3–82.1)	71.4 (57.1–79.8)					
• Min-max	35.7–96.4	28.6–92.9				39.3–100	19–85.7					
Quality of life, item 34			0.0 (-1.0;0.5)	0.320				-0.5 (-1.0;0.0)	**0.044**		0.0 (-1.0;1.0) 0.545	
• Md (Q1-Q3)	4.0 (4–5)	4.0 (2–6)				4.0 (3–6)	4.0 (2.5–5)					
• Min-max	1–7	1–7				1–7	1–6					

*Md* Median, *Q* quartile, *CI* confidence interval, α: Cronbach’s Alpha, *PCQ-P* Patient climate questionnaire, *LSQ* Life satisfaction questionnaire, *ICC* Intraclass correlation coefficient, *LMM* Linear Mixed Models, ^a^ related-samples Hodges-Lehman median difference, ^b^Wilcoxon Signed rank test for differences within group, ^c^independent-samples Hodges-Lehman median difference ^d^Mann-Whitney *U*-test for comparing change over time between the intervention and control groups (i.e. we first investigated change over time within the groups and then compared this change between the intervention and control group (interaction effect)). For empowerment there are 1 missing in the intervention group and 2 in the control group, for disempowerment there are 2 missing in the control group, for total PCQ-P and Hospitality there are 1 missing in the intervention group, for Quality of everyday activities there are 3 missing in the intervention group and 1 in the control group, for Socio-economic situation there is 1 missing in the intervention group, and for item 34 there is 1 missing in the intervention group

### Effects on person-centered climate

The intervention group had higher scores on total scale at follow-up compared to baseline (p ≤ 0.001). All subscales – safety (*p* = 0.001), everydayness (*p* = 0.009) and hospitality (p ≤ 0.001) – were rated higher at follow-up compared to baseline. The control group had lower scores on total scale at follow-up compared to baseline (*p* = 0.002). All subscales – safety (*p* = 0.013), everydayness (*p* = 0.028) and hospitality (*p* ≤ 0.001) – were rated lower at follow-up compared to baseline. Change over time between groups was significant for total scale (*p* ≤ 0.001) and for the subscales safety, everydayness and hospitality (*p* ≤ 0.001); see Table 3.

### Effects on life satisfaction

The intervention group had higher scores on the factor quality of everyday activities at follow-up compared to baseline (*p* = 0.033). The control group had lower scores on the factor quality of everyday activities at follow-up compared to baseline (*p* = 0.012). The control group further had lower scores on item 34, the single-item measure of quality of life, at follow-up compared to baseline (*p* = 0.044). In the intervention group, no significant differences were found for item 34 between baseline and follow-up. Regarding physical symptoms, sickness impact and socio-economic situation, no significant differences were found in either the intervention or control group between baseline and follow-up. The factors quality of family relations and quality of close friend relations were not possible to analyze due to the large amount of internal missing data. Change over time between groups was significant for quality of everyday activities (*p* = 0.002). For the other factors, there were no differences in change over time between the groups; see Table 3.

### Effects of the intervention using GEE models

Using GEE models the results revealed significant interaction effects i.e. differences in changes over time between the two groups: for empowerment (*p* = 0.002), disempowerment (*p* = 0.012), all factors safety (*p* < 0.001), everydayness (*p* < 0.001), hospitality (*p* < 0.001) and the total value for PCQ-P (*p* < 0.001), as well as the factor quality of everyday activities (*p* < 0.001) in the LSQ; see Tables 4 and 5. For the other factors the interaction effect was non-significant. For the intervention group there were significant improvement over time for the factors empowerment (*p* = 0.006), safety (*p* < 0.001), everydayness (*p* = 0.006), hospitality (*p* < 0.001), total values for PCQ-P (*p* < 0.001) and for the factor quality of everyday activities (*p* = 0.006). For the control group there were significant decline at follow-up compared to baseline for the factors disempowerment (*p* = 0.036), safety (*p* = 0.003), everydayness (*p* = 0.015), hospitality (*p* < 0.001), total values for PCQ-P (*p* < 0.001) and for the factor quality of everyday activities (*p* = 0.023); Tables 4 and 5.

**Table 4:** Parameter estimates and 95 % CIs from GEE analyses, Empowerment and Person-centered Climate Questionnaire (PCQ).

	Empowerment				Person-centered climate						PCQ tot value	
	Empowerment		Disempowerment		Safety		Everydayness		Hospitality			
	B	*P*-value	B	*P*-value	B	*P*-value	B	*P*-value	B	*P*-value	B	*P*-value
GEE Model												
Interaction time*group	-5.04 (-8.26;-1.81)	**0.002**	2.16 (0.47;3.85)	**0.012**	-7.39 (-10.63;-4.15)	**<0.001**	-3.88 (-6.00;-1.77)	**<0.001**	-5.78 (-7.45;-4.10)	**<0.001**	-16.67 (-22.43;-10.91)	**<0.001**
Mean differences												
Intervention group baseline-follow-up	-3.12 (-5.32;-0.91)	**0.006**	0.86 (-0.32;2.04)	0.153	-3.54 (-5.52;-1.57)	**<0.001**	-1.77 (-3.04;-0.50)	**0.006**	-2.94 (-4.12;-1.76)	**<0.001**	-7.87 (-11.31;-4.43)	**<0.001**
Control group baseline–follow-up	1.92 (-0.43;4.27)	0.110	-1.30 (-2.51;-0.09)	**0.036**	3.85 (1.28;6.41)	**0.003**	2.12 (0.42;3.82)	**0.015**	2.84 (1.65;4.02)	**<0.001**	8.80 (4.18;13.42)	**<0.001**

*CI* confidence interval, boldface text indicate statistically significant values, *GEE* Generalized estimating equation

**Table 5:** Parameter estimates and 95 % CIs from GEE analyses, Life Satisfaction Questionnaire (LSQ)

	LSQ	
	Quality of everyday activities	
	B	*P*-value
GEE Model		
Interaction time*group	-25.88 (-40.30;-11.46)	**<0.001**
Mean differences		
Intervention group baseline-follow-up	-13.39 (-22.97;-3.82)	**0.006**
Control group baseline–follow-up	12.49 (1.70;23.27)	**0.023**

*CI* confidence interval, boldface text indicate statistically significant values, *GEE* Generalized estimating equation

## Discussion

The present study aimed to evaluate effects on empowerment, person-centered climate and life satisfaction among older people living in residential facilities before and after a caregiver intervention concerning the Swedish national fundamental values. Results revealed that the caregiver intervention improved residents’ self-ratings of empowerment, person-centered climate and life satisfaction regarding the factor quality of everyday activities in the intervention group, while the control group had lower scores on person-centered climate and quality of everyday activities over time.

The positive intervention outcomes concerning empowerment may be due to the fact that residents had the opportunity, through the assignment to the intervention group, to engage in a dialogue with caregivers about their perceptions and experiences of the Swedish national fundamental values. For person-centered care it is essential to develop a clear picture of what the person values in life [40]. The person’s narrative is essential to person-centered care and therefore organizations must make more room for dialogue, through which staff can become acquainted with residents’ narratives. This requires that caregivers routinely invite persons to talk about their general situation, listen and respond to them. Moreover, it is important that this dialogue be allowed to take time [41]. Residents assigned to the intervention group had the opportunity to talk about their daily life and further to have a dialogue about it with caregivers. This approach on the part of caregivers is person-centered, as it helps residents gain power, influence and opportunities. A person-centered approach empowers individuals, and in the present study, the empowered individuals were residents at residential facilities.

Empowering care also promotes well-being [6]. The Swedish fundamental values state that well-being requires respect for the value of meaningfulness [4]. In the present study, meaningfulness was measured using the factor “quality of everyday activities.” To measure this, residents assessed to what extent their daily activities during the past week had been fun, interesting, creative, autonomous, useful and meaningful. In the intervention group, “quality of everyday activities” improved. In the Swedish national fundamental values, it is further stated that well-being requires respect for the value of safety [4]. In the present study, safety was measured using the factor “safety” in the Person-centered climate questionnaire, and had increased in the intervention group at follow-up. The present findings also support previous results showing that empowering care promotes well-being [6], as empowerment, meaningfulness and safety increased. Accordingly, we can conclude that an intervention concerning living in dignity and with a sense of well-being can promote empowering care, or vice versa that empowering care can promote living in dignity and with a sense of well-being for older people living in residential facilities.

Concerning life satisfaction, the factors “quality of family relations” and “quality of close friend relations” had a large amount of internal missing data in both groups, as residents stated that they had no family or friends. Previous research has also shown that residents, due to their advanced age, have few family members and friends [9, 15, 19]. Life satisfaction among residents is positively associated with family relationships [42]. Family and relationships are important for residents’ experience of quality of life [43, 44]. In the present study “life satisfaction” had decreased at follow-up in the control group. This decrease might be due to the absence of family and friends, although there was no similar decrease in “life satisfaction” in the intervention group, despite the absence of family and friends. This result may be explained by the increase in empowering care in the intervention group at follow-up, as empowering care is the most important predictor for quality of life among residents [7]. Because it is known that residents have few family members and friends due to their advanced age and that this negatively affects their experience of quality of life, it is essential to promote and develop empowering care in residential facilities, as such care is a predictor of quality of life.

In the intervention an interpretative approach was used and the positive intervention outcomes from the present study may be due to this approach. It has also been recommended to use a theoretical frame work for the intervention when implementing an intervention to change staff members’ practices in order to improve care of older people [45]. In an interpretative approach it is important that staff being actively involved in the process of creating a shared meaning for their work. Accordingly, it is important to take staff members’ ways of understanding their work as a point of departure [36, 37]. In the intervention, staff members’ understandings of living in dignity and with a sense of well-being were identified and a shared way of understanding this was created. Another important factor was that staff assigned to the intervention group identified residents’ ways of understanding living in dignity and with a sense of well-being. Staff members’ shared understanding and residents’ understanding of living in dignity and with a sense of well-being were then used as a point of departure for developing improvement plans aimed at increasing residents’ sense of dignity and well-being. Improvement plans were developed to increase meaningfulness in daily activities. The increase in “quality of everyday activities” in the intervention group at follow-up may be explained by these improvement plans. Further, in the intervention, self-reflection and dialogue were used as working methods. Reflection requires strategies and guidance by a mentor who makes the process meaningful, ties it to experiences and remains available throughout the learning process [46]. Accordingly, another important factor in the intervention was that the first author followed a protocol, guided all seminars, tied reflection to experiences and was further available throughout the intervention. The intention with the visits between the seminars and the visits after the eight seminars, i.e. the visits to the units every 2 weeks for two additional months to offer support to the staff as they put the improvement plan into practice, was to act as an external facilitator which is in line with several frameworks for implementation and research of factors that prevent or enable improvements in care practices [47, 48].

The results of the present study show that implementing the Swedish national fundamental values promotes empowering care, person-centered-climate and life satisfaction among residents. This means that the national fundamental values of living in dignity and with a sense of well-being, and their associated values (personal integrity, self-determination, participation, individualized care of good quality, a kind treatment, meaningfulness and safety) can provide support for caregivers when performing and developing care that promotes empowerment, person-centered climate and life satisfaction. These positive intervention outcomes indicate that it is essential that municipalities invest resources in putting the Swedish national fundamental values into practice in residential facilities for older people. It is further essential that managers at residential facilities, when they have the resources needed, prioritize putting the national fundamental values into practice, among other tasks. Moreover, it is vital that staff be given resources in the form of time to take part in reflective seminars on this process as well as that reflection be guided throughout the process. In the process of putting the national fundamental values into practice, it is also of major importance that residents and their views be included. When putting the national fundamental values into practice, with a view to increasing residents’ perceptions of living in dignity and with a sense of well-being, an intervention like the one carried out in the present study may be beneficial.

### Methodological limitations

The number of available participants in the intervention group was 156 and in the control group 144. Even, if we set out not to include units specialized for persons with dementia only 70 of 156 residents in the intervention group and 69 of 144 in the control group met the inclusion criterion, and of these only 43 in the intervention group and 37 in the control group participated in both data collection at baseline and follow-up. That less than half of the available participants could be asked to participate in the study was mainly due to the fact that they were diagnosed with dementia, despite this not living in a unit specialized for persons with dementia, or had reduced cognitive ability, with difficulties understanding the questions. This entails a limitation in external validity, as the results cannot be generalized to all residents living in the residential facilities as these include residents with cognitive impairment. Despite this limitation, it is important to examine the perspectives of residents whose cognitive ability is intact, as there is a lack of research in this area. Another limitation is that there were significant differences between the intervention and control groups at baseline and there were some drop-outs in both groups which are threats to internal validity, i.e. sample differences between the groups that could also cause the observed effect. We used complete/available case analyses [49] (also called a partial intention-to-treat analysis, all participants with complete data were analysed in the groups to which they were randomized) which may result in biased estimates. Another approach would have been to imputate values for the drop-outs. Reasons for drop-outs in both groups were deceased (7 intervention group; 6 control group), reduced cognitive ability (3 intervention group; 4 control group) and reduced general conditions (2 intervention group; 5 control group).

In the study, a power estimation indicated the need for 64 participants in each group. However, a weakness is that the power estimation was based on resident level and not unit level and on figures recommended for nursing intervention. According to Polit and Beck [31] a modest effect has been reported for most nursing interventions, and thereby with a power of 0.80 and alpha value of 0.05 the number of participants would be 64 in each group. In the study, randomization was performed after the residential facilities had been recruited, based on the inclusion and exclusion criteria, but before onset of data collection and the intervention. However, this was done because managers at the residential facilities needed to know early on whether they were in the intervention group, which required recruiting additional staff when the regular staff participated in seminars. Furthermore, the first author was not blind to which condition the participants were in. Another limitation is that only one post-test measure was conducted. It is important to know whether the effects of the intervention continued over time. Thus, including more post-test measures would have been preferable. The post-test measure was also carried out 2 months after the intervention was completed, which is a relatively short follow-up period. The reason for this, however, was that residents in residential facilities do not live long. Due to many analyses the risk of multiple testing bias needs to be taken into consideration, in total 11 factors or total scales has been tested. Regarding the person-centered climate, Cronbach’s alpha values were low for the factor hospitality, and regarding life satisfaction, they were low for the factor physical symptoms. Furthermore, the empowerment scale had not been used in a Swedish context before just tested for feasibility. Despite these limitations, the present study adds important knowledge about interventions that can increase empowerment, person-centered climate and life satisfaction among older people living in residential facilities.

## Conclusion

The present results indicate that when the Swedish national fundamental values of living in dignity and with a sense of well-being are put into practice, empowerment, person-centered climate and life satisfaction among older people living in residential facilities are increased when the intervention is carried out using an interpretative approach and guided throughout the process. Further, it would be interesting to investigate whether the intervention effects continued over time by performing the study on a larger scale and with more post-test measures as well as measuring the effects of the intervention on persons with cognitive impairment.

## Abbreviations

CI, Confidence Interval; EQ-5D, EuroQol 5-dimensions questionnaire; GEE, Generalized E stimating E quation; ICC, Intraclass Correlation coefficient; LSQ, Life Satisfaction Questionnaire; Md difference, Median difference; NA, Nurse Assistant; PCQ-P, Person-centered Climate Questionnaire - patient version; RN, Registered Nurse; SD, Standard Deviation 
